# The Passive Immunity Conferred by a Prophylactic Dose of Anti-tetanic Serum

**Published:** 1918-01

**Authors:** 


***The Passive Immunity Conferred by a Prophylactic Dose of Anti-tetanic Serum.*** By A. T. MacConkey, D. P. H., and Annie Homer. Abs. from the *Lancet*, Feb. 17, 1917.

With a view to determining, if possible, the duration of immunity conferred by anti-tetanic injections, the authors carried out a series of experiments on guinea-pigs.

They first sought the minimum dosage to render the animals immune for one week. This was found to be 1 unit for the guinea-pig, equivalent in the case of a man to about 250 U. S. A. units.

At the end of 10 days, the immunity was considerably diminished, and at the end of 2 weeks, it had almost disappeared, but sometimes sufficed to prevent death. At the end of 3 weeks the immunity had entirely disappeared.

The next tests made were to determine whether the term of immunity could be prolonged by increasing the preventive dose. Amounts up to 9 units were administered, but in no case was an animal immune at the end of 3 weeks. The dose was then increased to 40 units and toxin administered at the end of 2 weeks. Even this large dose did not render the animals completely immune for this short period. A dose of 250 units, however, gave a high degree of immunity, but yet not complete, for a period of 4 weeks.

The relative dose for man, however, would be quite impracticable.

The authors conclude that it is necessary, in order to maintain immunity, to repeat the usual protective doses, and quotes from a number of authorities to the effect that the injections should be repeated about once a week during the danger period.

